# The Quality of DNA Isolated from Processed Food and Feed via Different Extraction Procedures

**DOI:** 10.3390/molecules24061188

**Published:** 2019-03-26

**Authors:** Zora Piskata, Eliska Servusova, Vladimir Babak, Michaela Nesvadbova, Gabriela Borilova

**Affiliations:** 1 Department of Food and Feed Safety, Veterinary Research Institute, v.v.i., Hudcova 70, 62100 Brno, Czech Republic; servusova@vri.cz (E.S.); babak@vri.cz (V.B.); 2 Department of Meat Hygiene and Technology, University of Veterinary and Pharmaceutical Sciences Brno, Palackeho tr. 1946/1, 61242 Brno, Czech Republic; nesvadbovam@vfu.cz (M.N.); gborilova@vfu.cz (G.B.)

**Keywords:** DNA yield, meat products, polymerase chain reaction, DNA degradation, food analysis, food authenticity

## Abstract

The extraction of DNA is a critical step for species identification by PCR analysis in processed food and feed products. In this study, eight DNA extraction procedures were compared—DNeasy Blood and Tissue Kit, DNeasy mericon Food Kit, chemagic DNA Tissue 10 Kit, Food DNA Isolation Kit, UltraPrep Genomic DNA Food Mini Prep Kit, High Pure PCR Template Preparation Kit, phenol—chloroform extraction, and NucleoSpin Food—Using self-prepared samples from both raw and heat-processed and/or mechanically treated muscles and different types of meat products and pet food (pork, beef, and chicken). The yield, purity, and suitability of DNA for PCR amplification was evaluated. Additionally, comparisons between the effectiveness of various extraction methods were made with regard to price, and labor- and time-intensiveness. It was found that the DNeasy mericon Food Kit was the optimal choice for the extraction of DNA from raw muscle, heat-treated muscle, and homemade meat products from multiple and single species.

## 1. Introduction

Authenticity of species origin in food and feed products is important for law enforcement and for the protection of consumer health, as well as for economic and religious reasons. Total accuracy is not always guaranteed by labeling. For the identification of species in processed food products, an analytical approach must be used. Most analyses target proteins or DNA molecules which are extracted from tissues [1]. As the heating or canning process (121 °C, 15 min, 200 hPa) causes protein denaturation [2], DNA is a more suitable molecular marker for species authentication. In fact, DNA is also degraded into small fragments during the thermal process, but these are still detectable [3]. Moreover, DNA is largely unaffected by tissue source or sample damage [4,5]. However, fragment size is a limiting factor for the subsequent PCR [6]. Thus, DNA-based analytical methods represent a crucial approach for species identification in raw and highly processed meat products due to their high thermal stability and their very low detection limits. PCR and real-time PCR proved to be fast, reliable, and sensitive methods for species identification in foods and feeds with complex compositions [7,8,9,10,11,12,13,14]. Sequences of mitochondrial [15,16,17] and genomic DNA [18] were reported to be targeted as markers. The critical step in these approaches is the extraction of high-quality DNA in sufficiently large quantities from heterogeneous food matrices. Due to the fact that raw muscles are subjected to different treatments during the manufacturing process (high temperature, high pressure, addition of certain ingredients, etc.) which considerably influence the quality of DNA [19,20,21,22], it is necessary to individually optimize DNA isolation procedures for each type of food product. In addition, the chemical compounds present in food matrices (polysaccharides, proteins, collagen, polyphenols, fulvic acids, or lipids) may not be completely removed during the DNA extraction protocol and can affect the integrity of DNA or cause inhibition of subsequent PCR analysis [6]. Inhibitor compounds can interfere with PCR by decreasing or even completely inhibiting the activity of DNA polymerase [23]. Moreover, chemical reagents used in the isolation procedure may persist as contaminants, which can influence the purity of extracted DNA. Several authors already discussed various procedures for DNA extraction from raw and processed meat products [24,25] and raw beef [26], or compared the quality of DNA isolated from samples cooked at different temperatures [22,27]. A DNA extraction system should be effective in the sense that it should allow for the isolation of high quantities of good quality DNA with good PCR amplification ability; moreover, such a system should be affordable and should not impose heavy demands on labor and time.

The aim of this study was to determine the degree to which DNA is influenced by the technological processes used in the food industry (mechanical, thermal, chemical, and enzymatic treatments) using self-prepared samples from the muscle tissue of pigs, chickens, and cattle. We also intended to determine how the subsequent sample preparation and extraction procedure could affect the qualitative and quantitative parameters of DNA. It was also our objective to find suitable methods for homogenization and isolation of nucleic acids from samples obtained from raw, heat-treated, and/or mechanically processed muscle, and alternative types of meat products and animal feeds. Several extraction procedures for isolating DNA from meat products were tested and evaluated. The quality and yield of DNA were checked by measuring the absorbance (A) and determining the A260/A280 ratio using a spectrophotometer. Gel electrophoresis was performed to determine the level of DNA fragmentation caused by technological processing. Amplification ability was determined using PCR.

## 2. Results and Discussion

### 2.1. DNA Quantification and Purity

**Figure 1:**
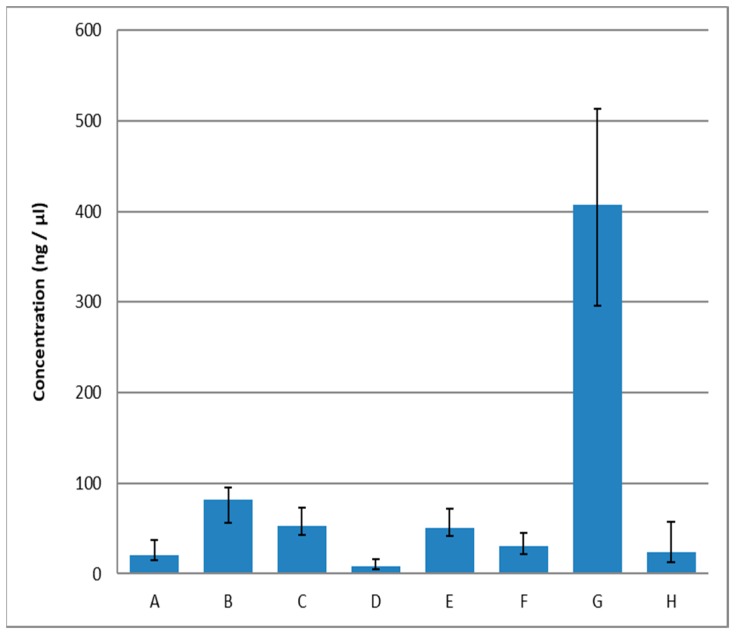
The average DNA concentrations obtained using the selected extraction methods A–H; A—DNeasy Blood and Tissue Kit (Qiagen), B—DNeasy mericon Food Kit (Qiagen), C—Chemagic DNA Tissue 10 Kit (PerkinElmer), D—Food DNA Isolation Kit (Norgen Biotek), E—UltraPrep Genomic DNA Food Mini Prep Kit (AHN-Bio), F—High Pure PCR Template Preparation Kit (Roche), G—phenol-chloroform extraction, H—NucleoSpin Food (Macherey-Nagel).

In processed products, DNA is exposed to heat and physical or chemical treatment, which can result in the degradation of DNA molecules [19,20,21]. The quality and yield of extracted DNA are critical for subsequent PCR analysis. In addition to the DNA quality and yield, it is also desirable that DNA should contain as few contaminants as possible. These would include proteins, polyphenols, polysaccharides, and any other PCR inhibitors [28]. The average DNA concentrations (ng/μL) obtained using the selected extraction techniques are presented in Figure 1. The lowest DNA yield was obtained using Food DNA Isolation Kit (Norgen Biotek) (kit D). The highest DNA concentrations were observed when using extraction method G (phenol—chloroform extraction). Nevertheless, these results could be misleading due to the particular procedure used for DNA extraction, which might have resulted in the presence of chemical reagents in the purified DNA samples because of a less thorough purification step. Such residual chemical contaminants could have caused an increase in absorbance (at 260 nm) of the DNA solution. Measured DNA concentrations are determined by the linear relationship between nucleic acid and absorbance in the ultraviolet (UV) region at 260-nm wavelength, and they are also dictated by the extinction coefficient. Consequently, A260 absorbance measurements are also sensitive to free nucleotides, ssDNAs and ssRNAs, as well as to some other organic contaminants such as chloroform and phenol [26], which can interfere with spectrophotometric analysis and may result in overestimation of the DNA yield. Musto (2011) reported that the denaturation of dsDNA to ssDNA, referred to as the hyperchromic effect, may in part be responsible for increased absorbance (at 260 nm) of DNA solutions [20].

**Figure 2:**
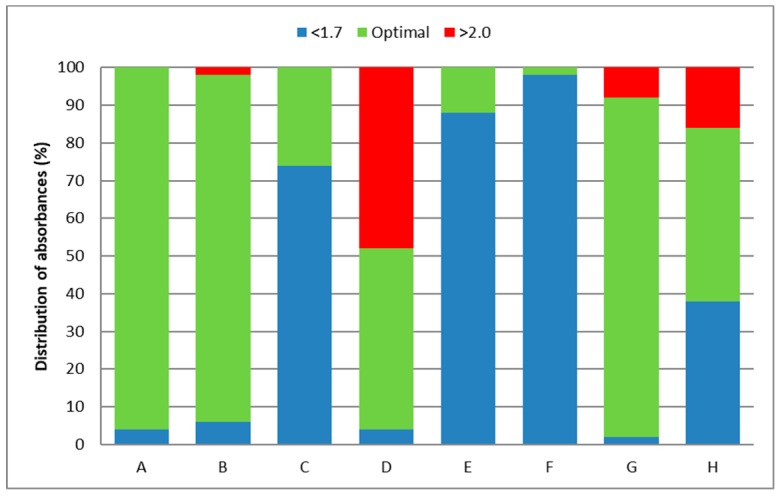
Distribution of A260/280 absorbance ratios; A—DNeasy Blood and Tissue Kit (Qiagen), B—DNeasy mericon Food Kit (Qiagen), C—Chemagic DNA Tissue 10 Kit (PerkinElmer), D—Food DNA Isolation Kit (Norgen Biotek), E—UltraPrep Genomic DNA Food Mini Prep Kit (AHN-Bio), F—High Pure PCR Template Preparation Kit (Roche), G—phenol-chloroform extraction, H—NucleoSpin Food (Macherey-Nagel).

In heat-treated muscle, the use of extraction method G (phenol-chloroform extraction) resulted in an amount of isolated DNA which was even higher than that from raw meat (Table 1). Similar findings were obtained by Reference [20], which claimed that the disruption of cell membranes during the heating process allowed more DNA to be released from the muscle cells. This contention was not confirmed when using other extraction methods. DNA yields of group 1 were higher, or at least comparable with DNA yields in groups 2, 3, and 4. The DNA was considered to be satisfactorily pure when the A260/A280 ratios were within the range of 1.7–2.0. Contamination of DNA with proteins usually reduces the A260/A280 ratio to values lower than 1.7 [29]. Residual impurities carried over from the DNA extraction procedure, such as phenol or ethanol, are also reported to reduce the A260/A280 ratio. The DNA yields differed significantly according to the type of extraction method; similarly, the purity of the extracts obtained did not always range within the above-mentioned ratio of A260/A280, i.e., 1.7–2.0 (Figure 2). 

The highest proportion of optimal absorbance values was obtained using extraction methods DNeasy Blood and Tissue Kit (Qiagen, Hilden, Germany) (A), DNeasy mericon Food Kit (Qiagen) (B), and G. By comparison, use of the F, E and C kits resulted in a high proportion of suboptimal values. Kit D gave a high score of more than 2.0. All extraction methods (A–H) gave similar results in terms of quality (purity ratios) for all four sample groups. We found extraction methods A, G and B to be preferable to kits High Pure PCR Template Preparation Kit (Roche, Basel, Switzerland) (F), UltraPrep Genomic DNA Food Mini Prep Kit (AHN-Bio) (E), and chemagic DNA Tissue 10 Kit (PerkinElmer) (C), because the latter kits showed a great deal of suboptimal absorption. Kit D gave a high proportion of high absorbance values (>2.0) for all sample types; however, with NucleoSpin Food (Macherey-Nagel, Düren, Germany) (kit H), this was only observed for samples of group 1. The purity of DNA was found to be highest in group 1 samples (raw meat), because most of the samples were in the optimal range of A260/A280 with the use of all tested extraction methods, except kit H.

### 2.2. DNA Fragmentation and PCR Amplification

The data described above provide information about the approximate yield and purity of the isolated DNA, but not about the potential fragmentation occurring during the technological processes used in the food industry. Another qualitative parameter of isolated DNA is, therefore, its integrity. DNA extracted from processed food is assumed to be fragmented [3], but the degree of fragmentation may vary; thus, the result on the gel is not a sharp band, but instead appears as a greatly expanded smear. When comparing different methods of isolation, one which gives a more compact band and larger molecular weights is better. Regardless of the extraction method, a considerable level of DNA degradation was observed in all samples. Some extraction methods lead to a higher fragmentation of DNA, which is revealed as a smudge across the lane without distinct maxima. Depending on a particular sample, some methods show low yields, as observed in almost all samples using methods D, E, and G. In samples which were heat-processed (100 °C and 120 °C), low yields were detected with almost all extraction approaches. The typical smear pattern of nucleic acid degradation of DNA extracted from heat-treated meats was observed also by Reference [22]. These data showed that the yield and integrity of DNA obtained from highly processed products were influenced by the type of processing. The suitability of DNA for PCR amplification was checked according to Reference [30]. The results of PCR amplification of DNA extracted from meat products demonstrated that there are measurable differences in the performance of each method (Table 2). Overall, the used extraction methods are comparable in terms of the PCR amplificability of DNA, with the exception of methods C and G which exhibited lower efficiencies (66.7% and 64.4%, respectively).

The results suggested that extraction methods G and C were least efficient for all sample types, whereas the other kits exhibited efficiencies that were similar to each other. However, none of the sample groups satisfied the null hypothesis of equal efficiency for all methods (*p* > 0.05, exact test for contingency tables). Apart from method G, the highest degree of PCR amplification was demonstrated in group 1 (raw meat), followed by groups 2 and 3. The samples in group 4 (feed samples) were less successfully amplified, probably due to the technology involved in production (granulation/pelletizing, canning) or the presence of other substances that can cause PCR inhibition. These observations suggest that a more sophisticated extraction technique should be developed for such samples. DNA extracted from heat-treated samples in group 2 exhibited bands of varying intensity in agarose gel electrophoresis (the higher the temperature used during the thermal process, the weaker the signal), indicating that some degree of DNA degradation occurred during the technological processing. This finding is in accordance with the results of References [20,27,31,32], where DNA was degraded during the thermal process. A comparison between lean and fat pork muscle revealed no differences when using kits B, D, F, and H, whereas, when using extractions A, C, E, and G, better PCR amplification was observed in some samples containing fat pork muscle. A correlation analysis showed no relationship between concentration, absorbance (or frequency of optimal concentration), and the PCR amplificability of DNA, as evidenced, e.g., using method G (high DNA concentrations, optimal A260/A280 ratios, but poor PCR amplification).

### 2.3. Summary Statistical Evaluation

Six parameters were considered for the comprehensive statistical evaluation of individual extraction methods: medians of concentrations (Conc.), medians of absorbances (Absorb.), percentage absorbance in the range of 1.7–2.0 (% Opt. Absorb.), percentage of observed bands in relation to the expected number of bands (Effic.), expense (Cost, 1 = +++, 2 = ++, 3 = +) and laboriousness (Labor, 1 = +++, 2 = ++, 3 = +). These six columns are followed by six columns with the same headings, with underlined terms presenting the average order of individual parameters within a group, ordered starting from highest average value of the corresponding parameter. The weighted average column displays the weighted mean calculated from the preceding six columns. For each parameter, the following scale (expressing the importance) was chosen (Table 1): Conc. 0.2 (high concentrations are desirable), Absorb. 0.1 (high absorbances are desirable, but not very high; therefore, the weight is lower than that of the concentrations), % Opt. Absorb. 0.2 (it is desirable that the concentration is in the range of 1.7 to 2.0), Effic. 0.3 (we consider the bands to be the most important parameter; thus, it has the highest weight), Cost 0.1 (a lower price is desirable, but it is not a crucial parameter), Laboriousness 0.1 (lower laboriousness is desirable, but it is not a crucial parameter). The last column shows the resulting order within a group. Taking into account all monitored parameters, kits B and A achieved the best ratings in groups 1–2, kits B and G in group 3, and kits A and G reached the highest preference in group 4. It should be noted that the obtained results fulfilled the aims of the experiment under conditions used in our study.

## 3. Materials and Methods

### 3.1. Sample Preparation

Authentic fresh meat samples from pigs (*Sus scrofa domesticus*), chickens (*Gallus gallus*), and cattle (*Bos taurus*) were purchased at local markets or were provided by a local slaughterhouse. The processing methods involved in the preparation of the selected types of meat products were amended from manufacturer recipes according to our conditions. Model samples included single-species products, minced meat mixtures, and products containing a defined percentage of the muscle of the studied species (pork, chicken, and beef). Bearing in mind that the natural heterogeneity of tissue composition can affect the efficiency of DNA extraction, both lean and fat pork muscle were tested. The basic set included samples of both lean pork muscle (loin, 3.6 g/100 g of total fat) and fat pork muscle (belly, 28 g/100 g of total fat), chicken breast muscle (1.5 g/100 g), and sample mixtures in defined ratios which were subjected to three forms of processing: cooking (cutting and subsequent heat treatment at 70 °C for 10 min), preserving (cutting and subsequent heat treatment at 100 °C for 10 min), and canning (cutting and subsequent heat treatment at 121.1 °C for 10 min). Another set of model samples consisted of proprietary meat products: a heat-processed meat product (Vienna sausage-like), an uncooked meat product (Teewurst), and a durable non-heat-treated fermented meat product (Rothwurst Polican sausage). These meat products were manufactured according to the commercial recipes for meat and meat products (two variants of each type of raw meat product) in the Technology Research Laboratories at the University of Veterinary and Pharmaceutical Sciences Brno, Czech Republic. Additionally, two samples of commercial feeds with defined compositions—granules and canned food—were tested. The final tested set comprised 25 samples; the degree of processing is described in Table 3. For better evaluation, the heterogeneous group of samples was divided into four subgroups: (1) raw muscle (single-species or mixtures), (2) heat-treated muscle, (3) meat products, and (4) feed.

### 3.2. DNA Extraction

DNA was isolated in duplicate using eight extraction protocols. Some of these were designed for the extraction of DNA even from highly processed food products, including canned products and other complex food and feed matrices. Seven commercial kits (DNeasy Blood and Tissue Kit (Qiagen, Hilden, Germany), DNeasy mericon Food kit (Qiagen, Hilden, Germany), Food DNA Isolation Kit (Norgen Biotek, Thorold, ON, Canada), UltraPrep Genomic DNA Food Mini Prep kit (AHN-Bio, Nordhausen, Germany), High Pure PCR Template Preparation Kit (Roche, Basel, Switzerland) and NucleoSpin Food (Machery-Nagel, Düren, Germany), chemagic DNA Tissue 10 kit (PerkinElmer, MA, USA), and one in-house extraction method (phenol-chloroform extraction) were used. The extraction procedures were performed according to the protocols supplied by the manufacturers or, in the case of the in-house extraction method, according to the validated laboratory protocol.

### 3.3. DNA Quantification and Purity

The quality of extracted DNA was determined by measuring concentration and purity using a UV spectrophotometer (NanoDrop™ 1000, Thermo Fisher Scientific, Waltham, MA, USA). DNA extracts were quantified by measuring the absorbance at 260 nm (A260) and 280 nm (A280). DNA purities were estimated by calculating the A260/A280 ratios. Samples calculated to have A260/A280 ratios of 1.7–2.0 were assumed to be pure samples, free from protein and other contamination. Every sample was measured three times. Instrument calibration was performed using the elution buffer. Measurement was done at room temperature following sufficient mixing of all samples.

### 3.4. DNA Fragmentation and PCR Amplification

To verify the degree of DNA integrity, which is usually disrupted in processed products, agarose gel electrophoresis was performed to determine DNA fragmentation. To check the suitability of the extracted DNA for subsequent PCR analysis, primers amplifying fragments of defined sizes (cattle—274 bp, pig—398 bp, chicken—227 bp) according to Reference [30] were tested. The presence or absence of bands on agarose gels was scored (+/−/0), and the data were expressed as DNA band positivity in relation to the total number of cases.

### 3.5. Statistical Analysis

To achieve normal distribution and to stabilize the variance, DNA concentration values from each group of samples were transformed using Box-Cox transformation. In the next step, data were analyzed using two-way ANOVA (factors: kit, sample) followed by the Tukey honestly significant difference (HSD) post hoc test. Distribution of the A260/A280 absorbance ratios and numbers of detected bands for each group of samples were evaluated using the exact test for contingency tables. For this study, *p*-values lower than 0.05 were considered statistically significant. Data analysis was performed using the statistical software Statistica 13.2 (StatSoft, Inc., Tulsa, OK, USA) and GraphPad Prism 5.04 (GraphPad Software, Inc., San Diego, CA, USA).

## 4. Conclusions

In the present study, the efficiency of eight methods for DNA extraction from food and feed with different compositions and subjected to different technological processing were assessed and compared. To determine the effectiveness of individual isolation procedures, several parameters that define the quality and quantity of isolated DNA (DNA concentration, A260/280 absorbance ratio, fragmentation of DNA, PCR amplification) were evaluated. Furthermore, a comparison of the effectiveness of the various kits with regard to cost, labor-intensiveness, and time-intensiveness was performed. The DNeasy mericon Food kit (Qiagen) appears to be the optimal choice for the extraction of DNA from raw muscle, heat-treated muscle, and homemade meat products of a single species or mixtures. Technological processing significantly affected the yield and quality of isolated DNA molecules, as well as the ability of DNA to undergo PCR amplification. The best results were obtained in group 1 (raw muscle), as expected. Correlation analysis showed no relationship between concentration, A260/A280 ratio, and the ability to undergo PCR amplification. It can be concluded that only PCR analysis can definitively establish the quality of the isolated DNA molecules.

## Figures and Tables

**Table 1:** Comprehensive evaluation of individual extraction methods; A—DNeasy Blood and Tissue Kit (Qiagen), B—DNeasy mericon Food Kit (Qiagen), C—Chemagic DNA Tissue 10 Kit (PerkinElmer), D—Food DNA Isolation Kit (Norgen Biotek), E—UltraPrep Genomic DNA Food Mini Prep Kit (AHN-Bio), F—High Pure PCR Template Preparation Kit (Roche), G—phenol-chloroform extraction, H—NucleoSpin Food (Macherey-Nagel).

Group	Extraction	Conc	Absorb	% Opt. Abs.	Effic. (%)	Cost	Laborious	Conc	Absorb	% Opt. Abs.	Effic. (%)	Cost	Laborious	Weighted Average *	Order
1	A	39.90	1.93	100	100	2	3	6	3	2	3.5	3.5	3.5	3.65	2
1	B	97.50	1.79	100	100	1	3	3	5	2	3.5	7	3.5	3.6	1
1	C	74.30	1.68	50	83.3	2	2	4	6	4.5	7	3.5	7	5.45	7
1	D	18.05	2.01	50	100	1	3	8	2	4.5	3.5	7	3.5	4.8	5
1	E	51.20	1.62	25	100	2	3	5	7	7	3.5	3.5	3.5	4.85	6
1	F	35.05	1.31	0	100	2	3	7	8	8	3.5	3.5	3.5	5.55	8
1	G	353.45	1.85	100	66.7	3	1	1	4	2	8	1	8	4.3	4
1	H	133.80	2.04	37.5	100	1	3	2	1	6	3.5	7	3.5	3.8	3
2	A	22.70	1.90	95.8	94.4	2	3	6	3	1	3	3.5	3.5	3.3	2
2	B	68.50	1.79	91.7	100	1	3	2	4	2	1	7	3.5	2.55	1
2	C	50.70	1.66	37.5	77.8	2	2	3	5	5	7	3.5	7	5.25	6
2	D	9.85	2.00	54.2	94.4	1	3	8	1	4	3	7	3.5	4.45	4
2	E	44.80	1.53	8.3	94.4	2	3	4	7	7	3	3.5	3.5	4.5	5
2	F	29.30	1.23	0	88.9	2	3	5	8	8	5.5	3.5	3.5	5.75	7
2	G	501.50	1.98	83.3	66.7	3	1	1	2	3	8	1	8	4.3	3
2	H	14.70	1.64	33.3	88.9	1	3	7	6	6	5.5	7	3.5	5.9	8
3	A	17.40	1.79	92.9	75	2	3	7	3	2.5	3	3.5	3.5	3.8	3
3	B	89.50	1.78	100	75	1	3	2	4	1	3	7	3.5	2.95	1
3	C	45.75	1.55	0	56.3	2	2	4	7	8	8	3.5	7	6.55	8
3	D	4.95	2.01	42.9	68.8	1	3	8	1	5	6.5	7	3.5	5.7	7
3	E	53.80	1.57	14.3	75	2	3	3	6	6	3	3.5	3.5	4	4
3	F	34.80	1.30	7.1	75	2	3	5	8	7	3	3.5	3.5	4.8	6
3	G	268.25	1.80	92.9	68.8	3	1	1	2	2.5	6.5	1	8	3.75	2
3	H	24.40	1.73	71.4	75	1	3	6	5	4	3	7	3.5	4.45	5
4	A	11.60	1.85	100	60	2	3	6	2	1.5	1.5	3.5	3.5	2.85	1
4	B	28.30	1.69	50	40	1	3	5	4	3.5	5.5	7	3.5	4.8	5
4	C	129.50	1.43	0	40	2	2	2	7	7	5.5	3.5	7	5.2	6
4	D	4.30	1.97	25	40	1	3	8	1	5	5.5	7	3.5	5.4	7
4	E	83.95	1.55	0	60	2	3	3	6	7	1.5	3.5	3.5	3.75	3
4	F	8.65	1.23	0	40	2	3	7	8	7	5.5	3.5	3.5	5.95	8
4	G	462.05	1.81	100	40	3	1	1	3	1.5	5.5	1	8	3.35	2
4	H	55.00	1.62	50	40	1	3	4	5	3.5	5.5	7	3.5	4.7	4

* Weights: 0.2 (Conc—Concentration), 0.1 (Absorb—Absorbance), 0.2 (%Opt. Abs.—Optimal absorbance), 0.3 (Effic—Efficiency), 0.1 (Cost), 0.1 (Laborious—laboriousness).

**Table 2:** Frequency analysis of the number of detected bands by PCR; A—DNeasy Blood and Tissue Kit (Qiagen), B—DNeasy mericon Food Kit (Qiagen), C—Chemagic DNA Tissue 10 Kit (PerkinElmer), D—Food DNA Isolation Kit (Norgen Biotek), E—UltraPrep Genomic DNA Food Mini Prep Kit (AHN-Bio), F—High Pure PCR Template Preparation Kit (Roche), G—phenol-chloroform extraction, H—NucleoSpin Food (Macherey-Nagel).

Extraction Method	Expected Bands	Detected Bands	%
A	45	38	84.4
B	45	38	84.4
C	45	30	66.7
D	45	36	80.0
E	45	38	84.4
F	45	36	80.0
G	45	29	64.4
H	45	36	80.0
Total	360	281	78.1

**Table 3:** Samples tested in the study.

No.	Chicken (%)	Pork Loin (%)	Pork Belly (%)	Pork Backfat (%)	Beef (%)	Processing Type	Group
							
1	0	100	0	0	0	raw	1
2	0	100	0	0	0	70 °C/10 min	2
3	0	100	0	0	0	100 °C/10 min	2
4	0	100	0	0	0	121.1 °C/10 min	2
5	90	10	0	0	0	raw	1
6	90	10	0	0	0	70 °C/10 min	2
7	90	10	0	0	0	100 °C/10 min	2
8	90	10	0	0	0	121.1 °C/10 min	2
9	0	0	100	0	0	raw	1
10	0	0	100	0	0	70 °C/10 min	2
11	0	0	100	0	0	100 °C/10 min	2
12	0	0	100	0	0	121.1 °C/10 min	2
13	90	0	10	0	0	raw	1
14	90	0	10	0	0	70 °C/10 min	2
15	90	0	10	0	0	100 °C/10 min	2
16	90	0	10	0	0	121.1 °C/10 min	2
17	12	50	0	10	28	Vienna sausage-like (type 1)	3
18	31	31	0	10	28	Vienna sausage-like (type 2)	3
19	50	12	0	10	28	Vienna sausage-like (type 3)	3
20	0	20	0	80	0	Teewurst (type 1)	3
21	0	40	0	60	0	Teewurst (type 2)	3
22	0	38	0	33	29	Fermented sausage (type 1)	3
23	8	32	0	30	30	Fermented sausage (type 2)	3
24	+	+	nd *	nd	-	Pet food (granules)	4
25	43	35	nd	nd	20	Pet food (canned)	4

* Not defined.
